# FIP1L1-PDGFRA-Positive Chronic Eosinophilic Leukemia: A Low-Burden Disease with Dramatic Response to Imatinib - A Report of 5 Cases from South India

**DOI:** 10.4274/Tjh.2013.0086

**Published:** 2014-03-05

**Authors:** Anıl N. Kumar, Vishwanath Sathyanarayanan, Visweswariah Lakshmi Devi, Namratha N. Rajkumar, Umesh Das, Sarjana Dutt, Lakshmaiah K Chinnagiriyappa

**Affiliations:** 1 Kidwai Memorial Institute of Oncology, Department of Medical Oncology, Karnataka, India; 2 Kidwai Memorial Institute of Oncology, Department of Pathology, Karnataka, India; 3 Oncquest Laboratories Ltd., New Delhi, India

**Keywords:** PDGFRA, Chronic eosinophilic Leukemia, Imatinib, India

## Abstract

**Objective:** Eosinophilia associated with FIP1L1-PDGFRA rearrangement represents a subset of chronic eosinophilic leukemia and affected patients are sensitive to imatinib treatment. This study was undertaken to learn the prevalence and associated clinicopathologic and genetic features of FIP1L1-PDGFRA rearrangement in a cohort of 26 adult patients presenting with profound eosinophilia (>1.5x10^9^/L).

**Materials and Methods:** Reverse-transcriptase polymerase chain reaction and gel electrophoresis were used for the detection of FIP1L1-PDGFRA rearrangement.

**Results:** Five male patients with splenomegaly carried the FIP1L1-PDGFRA gene rearrangement. All patients achieved complete hematological response within 4 weeks of starting imatinib. One patient had previous deep vein thrombosis and 1 patient had cardiomyopathy, which improved with steroids and imatinib. Conventional cytogenetics was normal in all these patients. No primary resistance to imatinib was noted.

**Conclusion:** This study indicates the need to do the FIP1L1-PDGFRA assay in patients with hypereosinophilic syndrome. Prompt treatment of this condition with imatinib can lead to complete hematological response and resolution of the organ damage that can be seen in this setting.

## Abstract (tr)

**Amaç:** FIP1L1-PDGFRA rearranjmanı ilişkili eozinofili, kronik eozinofilik löseminin bir alt grubunu temsil eder ve etkilenen hastalar imatinib tedavisine duyarlıdır. Bu çalışma, şiddetli eozinofili ile başvuran 26 erişkin hastadan oluşan bir grupta FIP1L1-PDGFRA rearranjmanının klinikopatolojik ve genetik yaygınlığını, ve özelliklerini öğrenmek için yapıldı.

**Gereç ve Yöntemler:** FIP1L1-PDGFRA rearranjmanının tespiti için revers-transkriptaz polimeraz zincir reaksiyonu ve jel elektroforezi kullanıldı.

**Bulgular:** Splenomegaliye sahip beş erkek hasta FIP1L1-PDGFRA gen yeniden düzenlenmesini taşıyordu. İmatinib başlangıcından 4 hafta içinde tüm hastalarda tam hematolojik yanıt elde etti. Bir hastada önceden var olan derin ven trombozu ve 1 hastada steroid ve imatinib ile düzelen kardiyomiyopati vardı. Tüm bu hastalarda konvansiyonel sitogenetik normaldi. İmatinibe karşı birincil direnç kaydedilmedi.

**Sonuç:** Bu çalışma hipereozinofilik sendromlu hastalarda FIP1L1-PDGFRA testinin yapılması gerektiğini gösterir. Imatinib ile bu durumun acil tedavisi tam hematolojik yanıta ve bu durumda görülebilecek organ hasarının engellenmesine yol açabilir.

## INTRODUCTION

Hypereosinophilic syndrome (HES) is an uncommon disorder with persistent eosinophilia and multiple organ dysfunction due to eosinophilic infiltration [1]. The spectrum of clinical manifestations are variable and patients may be asymptomatic or may have endomyocardial fibrosis or restrictive lung disease. Clonal or neoplastic eosinophilia is defined as eosinophilia originating from the malignant clone in hematopoietic stem cells and myeloid neoplasms. Myeloproliferative neoplasm with eosinophilia and platelet-derived growth factor receptor-alpha gene and Fip1-like 1 gene mutation (FIP1L1-PDGFRA; F/P) is a low-burden disease with dramatic response to imatinib therapy. Various classifications over the last 20 years, and especially from the Year 2011 Working Conference on Eosinophil Disorders and Syndromes, have tried to give us a better understanding of the pathophysiology and management of this rare but clinically important and treatable hematological malignancy [1]. There are numerous reports indicating an ongoing effort for treatment of this condition in patients of different genetic backgrounds, such as those of Özbalcı et al. from Turkey [2], Loules et al. from Greece [3], Arai et al. from Japan [4], and Helbig et al. from Poland [5]. There are very few reports from India, including those of Kumar et al. [6], Sreedhar Babu et al. [7], and Arora [8]. Hence, we undertook this study to learn the clinical profile and outcome in our region. 

## MATERIALS AND METHODS

**Figure 1:**
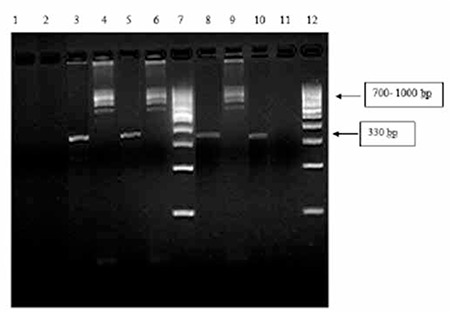
PCR analysis of FIP1L1-PDGFRA fusion isolated from one of our patients.

This study was done at the Kidwai Memorial Institute of Oncology, Bangalore, Karnataka, India, a tertiary care oncology center in southern India. During the period from January 2008 to December 2011, among the patients attending our hematology clinic, cases of eosinophilia were reviewed. he clinical profile, history, physical examination findings, hemogram investigations and metabolic panels were reviewed. Patients underwent bone marrow aspiration and biopsy when required. Where indicated in the investigation, F/P fusion assay was done by Oncquest Laboratory, New Delhi, India, using the protocol described below (Figure 1). Informed consent was obtained.

**RNA Isolation**

RNA was extracted from bone marrow or peripheral blood using the RNeasy Mini Kit supplied by QIAGEN as per the manufacturer’s instructions. RNA was quantified using a SmartSpec 3000 spectrophotometer (Bio-Rad). The quality of RNA was ascertained by resolving it on 1.2% formaldehyde MOPS gel. The purified RNA was stored at -80 °C until further processing. 

**cDNA Preparation**

cDNA was prepared using the RevertAid First Strand cDNA Kit supplied by MBI Fermentas. First, 1 µg of total RNA was taken in a thin-walled, 0.2 mL tube and the volume was made up to 12 µL with nuclease-free water. The RNA was denatured by incubating at 65 °C for 5 min in a Dyad Peltier Thermal Cycler (Bio-Rad). The denatured RNA was snap-chilled by keeping it on ice for 2 to 4 min, and then 1 µL of dNTPs, 1 µL of random hexamer primer, 4 µL of 5X RT buffer, and 1 µL of RNase inhibitor was added to the tube. This was incubated at 20 °C for 4 min in the thermal cycler; 1 µL of reverse transcriptase was then added and this was incubated at 42 °C for 50 min. The cDNA hybrid was denatured by incubating at 93 °C for 5 min. The cDNA was stored at -80 °C in a deep freezer until further processing.

**PCR and Analysis**

Fusion of FIP1L1 to PDGFRA was analyzed in an end-point polymerase chain reaction (PCR) using the following primers: FIP1L1 FP (5’ ACCTGGTGCTGATCTTTCTGAT 3’) and PDGFRA RP (5’ TGAGAGCTTGTTTTTCACTGGA 3’). 

Briefly, 3 µL of cDNA was taken in a 0.2 mL PCR tube. To this was added 2 µL each of 5 µM forward and reverse primers (Sigma), 0.5 µL of 10 mM dNTP mix, 2.5 µL of 10X buffer, and 0.5 µL of DyNAzyme II DNA polymerase (all from Finnzymes). The final reaction volume was made up to 25 µL with nuclease-free water (Ambion). After an initial denaturation at 94 °C for 3 min, the PCR was run for 45 cycles with these conditions: 94 °C for 30 s; 60 °C for 30 s; and 

72 °C for 30 s followed by a final extension at 72 °C for 2 min. PCR directed at the amplification of a housekeeping gene was used as an internal control to ensure mRNA quality. A “no template control” (NTC) was used to detect the incidence of false-positive reactions. All reactions were carried out in a Dyad Peltier Thermal Cycler (Bio-Rad). The amplicon was resolved on 2% agarose gel to ascertain the specificity of amplification. The resolved PCR products were visualized under UV illumination and documented on a gel doc system (UVP). Presence of the FIP1L1-PDGFRA deletion mutation resulted in varying amplicons of 700-1000 bp. 

1 2 3 4 5 6 7 8 9 10 11 12

Lanes 1 and 2: NTC for housekeeping control and FIP1L1.

Lanes 3 and 4: Amplicon for housekeeping gene (330 bp) and FIP1L1-PDGFRA (700-1000 bp) of patient A.

Lanes 5 and 6: Amplicon for housekeeping gene (330 bp) and FIP1L1-PDGFRA (700-1000 bp) of patient B.

Lane 7: 100-bp marker.

Lanes 8 and 9: Amplicon for housekeeping gene (330 bp) and FIP1L1-PDGFRA (700-1000 bp) of positive control.

Lanes 10 and 11: Amplicon for housekeeping gene (330 bp) and FIP1L1-PDGFRA (700-1000 bp) of negative control.

Lane 12: 100-bp marker.

## RESULTS

During the time period mentioned, 26 patients had analysis for the F/P mutation, with 5 positive cases representing 19.2% of total cases. This is probably a falsely high figure and does not reflect the true incidence, as our hospital is a reference center for oncology. All 5 patients were male. The median age was 43.8 years (range: 21-52). Palpable splenomegaly was seen in all patients, which was confirmed by ultrasound. 

The clinical and laboratory parameters are included in Table 1. 

Bone marrow showed a marked increase in eosinophils. These were mostly mature; however, eosinophils with sparse purple granules, vacuolation, and hyposegmented forms were seen. Few or no blasts and an increase in masT-cells were seen. Conventional cytogenetics, done in all cases, showed normal karyotypes. 

All patients were started on imatinib at 100 mg or 400 mg per day as per the physician’s preference. All patients achieved complete hematological response within 4 weeks. One patient was symptomatic with pedal edema and dyspnea. Two-dimensional echocardiogram revealed cardiomyopathy with left ventricular thrombus. The patient showed significant improvement in symptoms with imatinib and steroids. Another patient had pre-existing deep vein thrombosis, which also resolved with imatinib therapy.

## DISCUSSION

The Year 2011 Working Conference on Eosinophil Disorders and Syndromes (Vienna, Austria) was a multispecialty discussion held in order to formulate a consensus statement on the classification of eosinophil disorders and related syndromes [1]. 

HES is characterized by persistent eosinophilia and is associated with damage to multiple organs. Hardy and Anderson first described this entity in 1968 [9]. Later, in 1975, Chusid et al. defined the prerequisites for the diagnosis of HES [10] as follows: 

1. Absolute eosinophil count of greater than >1500/µL persisting for longer than 6 months. Outcomes of the Year 2011 Working Conference on Eosinophil Disorders and Syndromes suggested that at least 2 occasions with a minimum time interval of 4 weeks of eosinophilia can also be considered persistent [1]. 

2. No identifiable etiology for eosinophilia. 

3. Patients must have signs and symptoms of organ involvement. 

Other causes of eosinophilia, like familial eosinophilia, and acquired causes that are subcategorized as secondary, clonal, and idiopathic eosinophilia should be considered [1].

The rational algorithm for a patient with eosinophilia includes peripheral blood smear to rule out an underlying myeloid malignancy (eg., circulating blasts and dysplastic cells) and serum tryptase level. Peripheral blood testing for the F/P mutation should also be done as an initial test. The next step would be to do a bone marrow aspiration to rule out myeloid neoplasm or chronic eosinophilic leukemia not otherwise specified (CEL-NOS) [11,12]. 

In CEL-NOS, persistent eosinophilia associated with blasts in the peripheral blood or bone marrow of 20% or clonality of the eosinophils must be proven [11]. A separate category has been made for myeloid and lymphoid neoplasms with eosinophilia and abnormalities of PDGFRA, PDGFRB, or FGFR1. In neoplasms with F/P mutation, the presentation is usually CEL and less often acute myeloid leukemia or T-lymphoblastic lymphoma [12].

After the tremendous success of imatinib therapy in BCR-ABL-positive chronic myelogenous leukemia, imatinib was tried in various myeloproliferative disorders and patients with eosinophilia were found to be particularly sensitive. A translational research effort led to the identification of a constitutively activated fusion tyrosine kinase on chromosome 4q12, derived from an interstitial deletion, that fuses with PDGFRA to an uncharacterized human gene, FIP1L1, first described by Cools et al. in 2003 [13]. They used a nested PCR approach, but our used method was reverse-transcriptase PCR.

There have been reports from around the world regarding the treatment of F/P-positive CEL with imatinib [2,3,4,5]. In most of the reports, all patients were male, except for 2 of 27 patients reported by Helbig et al. [5]. All patients had splenomegaly, which is also reflected in our series. All of our patients achieved complete hematologic response within 4 weeks, which is on par with the results of other studies. The optimal dose of imatinib has been a subject of debate. Successful treatment regimens have ranged from 100 mg/week to 400 mg/day [5,14]. The frequency of emergence of imatinib resistance in F/P-positive disease is currently unclear [15,16]. We observed no hematologic relapses among our patients, although our follow-up time was short. The concentration of imatinib required to inhibiT-cells transformed by FIP1L1-PDGFRA by 50% (IC50) was 3.2 nM, whereas the IC50 for BCR-ABL was 582 nM. Hence, complete remission even with low doses of imatinib is seen, which is also evident in our case series. One of our patients showed a resolution of cardiac complications similar to that reported by Arai et al. [4].

Few cases have been reported from India and ours is the largest reported Indian series. The patient reported by Arora [8] had a cytogenetic abnormality, t (1; 4) (q24; q35). In all other cases, diagnoses were made on clinical and morphological grounds and the F/P mutation was not analyzed. Due to this, the true incidence of CEL in India is not known.

## CONCLUSION

PDGFRA-positive myeloid neoplasm with eosinophilia is a rare disease and this study indicates the need to do the PDGFRA assay in patients with HES. A high index of suspicion among clinicians and awareness of this condition is essential for prompt initiation of therapy. The response is dramatic and can lead to complete hematological response and prevention or resolution of organ damage due to eosinophilic infiltration. 

## CONFLICT OF INTEREST STATEMENT

The authors of this paper have no conflicts of interest, including specific financial interests, relationships, and/ or affiliations relevant to the subject matter or materials included.

## Figures and Tables

**Table 1:**
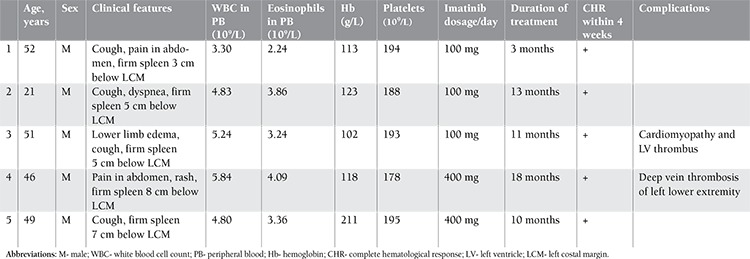
Clinical and laboratory profiles of 5 patients.
